# Experimental design, preprocessing, normalization and differential expression analysis of small RNA sequencing experiments

**DOI:** 10.1186/1758-907X-2-2

**Published:** 2011-02-28

**Authors:** Kevin P McCormick, Matthew R Willmann, Blake C Meyers

**Affiliations:** 1 Department of Plant and Soil Sciences and Delaware Biotechnology Institute, University of Delaware, Newark, DE 19711, USA; 2 Department of Biology, University of Pennsylvania, Philadelphia, PA 19104, USA

## Abstract

Prior to the advent of new, deep sequencing methods, small RNA (sRNA) discovery was dependent on Sanger sequencing, which was time-consuming and limited knowledge to only the most abundant sRNA. The innovation of large-scale, next-generation sequencing has exponentially increased knowledge of the biology, diversity and abundance of sRNA populations. In this review, we discuss issues involved in the design of sRNA sequencing experiments, including choosing a sequencing platform, inherent biases that affect sRNA measurements and replication. We outline the steps involved in preprocessing sRNA sequencing data and review both the principles behind and the current options for normalization. Finally, we discuss differential expression analysis in the absence and presence of biological replicates. While our focus is on sRNA sequencing experiments, many of the principles discussed are applicable to the sequencing of other RNA populations.

## Introduction

Deep sequencing technologies have revolutionized the field of genomics since their inception in 2000, when Lynx Therapeutics' Massively Parallel Signature Sequencing (MPSS; Lynx Therapeutics, Hayward, CA, USA) was described as a way to quantify messenger RNA (mRNA) populations [1]. MPSS allowed the parallel sequencing of 17- or 20-nucleotide (nt) signatures from hundreds of thousands of cloned RNA, but it has been made obsolete by newer systems enabling longer sequence reads with fewer biases. Next-generation sequencing has since been adapted to the study of a wide range of nucleic acid populations, including mRNA (RNA-seq) [2], small RNA (sRNA) [3], microRNA (miRNA)-directed mRNA cleavage sites (called parallel analysis of RNA ends (PARE), genome-wide mapping of uncapped transcripts (GMUCT) or degradome sequencing) [4-6], double-stranded RNA (dsRNA) [7,8], actively transcribing RNA (NET-seq) [9], translated mRNA [10], transcription factor DNA binding sites and histone modification sites (chromatin immunoprecipitation (ChIP)-seq) [11], methylated DNA (BS-seq) [12] and genomic DNA (DNA-seq) [13-15]. These applications vary with regard to the templates used, but they rely on the same sequencing technologies.

Prior to high-throughput sequencing, DNA microarrays were the predominant method of genome-wide transcriptional analysis. Microarrays have been used to quantify the levels of both known and unknown mRNA, alternative splicing products, translated mRNA and miRNA, as well as to detect miRNA cleavage sites, transcription factor binding sites, single-nucleotide polymorphisms and deletions. Now, however, high-throughput sequencing is often favored over microarrays for such experiments because sequencing avoids several problems encountered in microarray experiments. First, unlike microarrays, sequencing approaches do not require knowledge of the genome *a priori*, enabling any organism to be easily studied. Second, sequencing is not dependent on hybridization. Microarray data are obtained by hybridizing a labeled target to complementary DNA probes immobilized on a solid surface, and the strength of this hybridization is dependent on the base composition of the probe [16-20]. With arrays, it is possible for cross-hybridization to occur, such that the signal may come from sources besides the perfectly complementary intended target [17,18,21]. Sequencing, however, has a single-nucleotide resolution, which increases specificity and is far superior for certain applications, such as defining transcription factor binding sites to the probe-defined resolution of microarrays. Third, sequencing produces digital data by counting the number of copies of a particular sequence, enabling accurate determination of low-, middle- and high-abundance species. Because microarray data are based on the intensity of the fluorescence label at each spot on the hybridized array and intensity falls on a continuum, the data are analog. The disadvantage of this is that it is hard to accurately quantify signals at the two extremes: signals near the lower limit of detection [22-28] and those near the intensity saturation point [29,30]. The proper quantification of intensity also depends on accurate measurement of background levels, which is not an issue for digital data [31-33]. Although sequencing is free from these intrinsic experimental limitations, microarray experiments are cheaper (at the moment) and do not suffer from ligation biases (discussed below in the section "Library preparation and inherent biases").

Next-generation sequencing has proved to be a boon to the study of sRNA. Sequencing of individual sRNA clones by traditional Sanger sequencing was laborious and did not achieve a sufficient sequencing depth to detect rare species [34-39]. There are several biologically relevant and functionally diverse classes of sRNA of specific sizes and produced by different, genetically separable pathways. These include miRNA, small interfering RNA (siRNA) and the animal-specific Piwi-interacting RNA (piRNA, originally called repeat-associated siRNA or rasiRNA). miRNA are 19 to 25 nt long and originate from noncoding RNA called pri-miRNA that have extensive secondary structure [40]. miRNA posttranscriptionally silence non-self-targeted mRNA through imperfect base pairing, directing target cleavage [41,42] or translational inhibition [40,43].

The biogenesis of miRNA is in contrast to that of siRNA (20 to 24 nt), which are formed from long dsRNA [44-46]. siRNA can direct the cleavage of perfectly base-paired mRNA, including the RNA from which they originate [34,46]. Several subclasses of siRNA exist, which vary by name or by type in different organisms. In animals, siRNA are designated on the basis of their source: endogenous dsRNA (endo-siRNA, or esiRNA) and exogenous dsRNA (exo-siRNA) [47,48]. esiRNA are derived from long dsRNA made by RNA-dependent RNA polymerases (RDRs) from sense transcripts, pairing between convergent transcripts (sense and natural antisense transcripts) or long self-complementary RNA, while exo-siRNA come from RNA viruses. The *Caenorhabditis elegans *and plant literature distinguish primary siRNA, that is, those that are formed from the dsRNA that initiates a silencing event, from secondary siRNA, that is, those that are formed from the cleaved target mRNA and perpetuate and amplify silencing [49-52]. In plants, siRNA are also defined based on their origin and/or function and include heterochromatic siRNA (hc-siRNA, sometimes also referred to as rasiRNA), natural antisense transcript-derived siRNA (nat-siRNA), and *trans*-acting siRNA (ta-siRNA). hc-siRNA are 23- to 24-nt siRNA found in plants and *Schizosaccharomyces pombe *that direct methylation of DNA and histones, leading to transcriptional gene silencing, particularly in repeat regions [53-55]. A second subset of siRNA in plants, nat-siRNA, arise from the hybridization of sense transcripts with their naturally occurring antisense forms and subsequent cleavage [56]. siRNA derived from natural antisense transcripts are also found in animals, but are not always referred to as nat-siRNA [57-60]. ta-siRNA appear to be plant-specific and originate from noncoding RNA that are the targets of miRNA. Following miRNA cleavage, the cleavage products are made double-stranded and then chopped into 20- or 21-nt ta-siRNA. These ta-siRNA target non-self-targeted mRNA via imperfect base pairing for cleavage, similarly to miRNA [61-64].

The most recently identified major class of sRNA is the piRNA group, a 25- to 30-nt sRNA associated with the Piwi subclade of Argonaute family of proteins, and these sRNA have functions in the germline of animals [65-71]. All of these kinds of sRNA can be identified by generating sRNA sequencing libraries from size-selected populations of RNA that are approximately 18 to 30 nt long. Along with these biologically relevant sRNA, RNA degradation products, including fragments of transfer RNA (tRNA) and ribosomal RNA (rRNA), are also sequenced. Studies have found an abundance of specific tRNA-derived sRNA in *Saccharomyces cerevisiae*, *Arabidopsis *and human cells [72-74], at least some of which are Dicer cleavage products [73], and methionine tRNA, or tRNA^Met^, was associated with human Argonaute 2 protein, or Ago2, in human cells [75]. The finding by the Dutta laboratory [72] that some of these tRNA sequences, called tRNA-derived RNA fragments, have a biological function further suggests that new classes of and roles for sRNA will likely continue to be identified.

Sequencing can also be used to study sRNA targets. RNA-seq can directly quantify expression levels of mRNA that are targets of sRNA. High-throughput sequencing has recently been applied to the identification of miRNA cleavage sites, a method alternately called degradome sequencing [4], PARE [5] and GMUCT [6]. This approach is useful for identifying precise miRNA target sites because the fragment immediately downstream of the cleavage site will appear much more abundantly than any surrounding sequences produced by nonspecific decay. These methods will not detect the effects of miRNA on target translation, however. New approaches that combine immunopurification of polysomes (mRNA that are associated with ribosomes) with deep sequencing allow for the sequencing of RNA that are actively being translated and enable the detection of miRNA-mediated translational inhibition [10,76]. In contrast to miRNA, the target of hc-siRNA is chromatin, and hc-siRNA-induced DNA and histone methylation can be identified using BS-seq and ChIP-seq, respectively.

**Figure 1:**
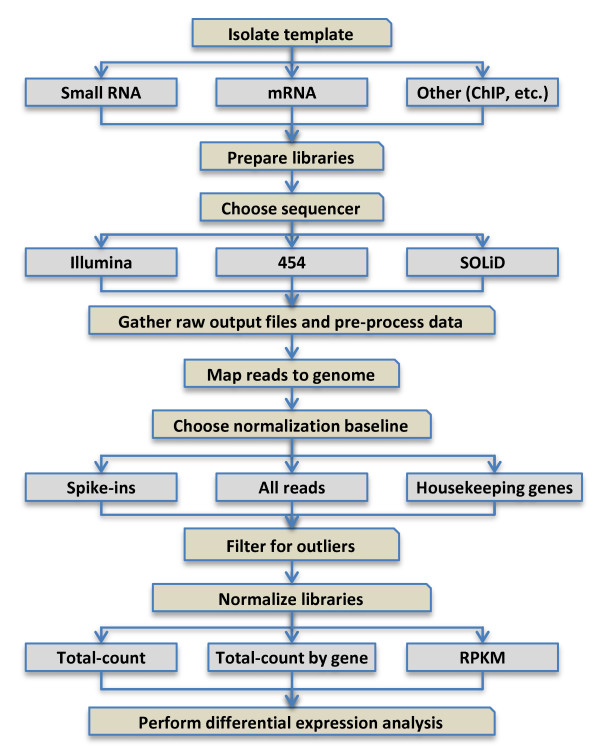
**Flowchart of typical data-handling steps for small RNA (sRNA) libraries**. Flowchart depicting the steps involved in creating, processing and normalizing next-generation sequencing libraries. In this article, we focus on sRNA data, but the methods for analyzing other RNA-based or even chromatin immunoprecipitation sequencing data are similar.

Next-generation sequencing data sets are similar to one another in several aspects, regardless of the technology or template used. In all cases, raw data files in the form of images must be preprocessed and normalized before they can be stored for analysis or visualization. The preprocessing of data comprises a series of steps that involve converting image files to raw sequences (also called "reads"), handling low-quality base calls, trimming adapters from raw sequencing reads, tabulating numbers of trimmed reads per distinct sequence and aligning these reads to a reference genome if available. Normalization, the process of comparing raw sequence counts against some common denominator, is a critical step when processing expression data of all types. Normalization removes technical artefacts arising from the method itself or from unintended variation, with the goal that differences remaining between samples are truly or predominantly biological in nature. Figure 1 demonstrates the flow of data for typical sequencing experiments.

In this review, we consider the design of sRNA sequencing experiments, the preprocessing and normalization of sequencing data and basic differential expression analysis. We discuss various approaches for normalizing sequencing data, starting with what has been learned from microarrays about the fundamentals of normalizing large-scale transcriptional data sets. Because the cost of sequencing is still somewhat high (although it is dropping rapidly), many experiments do not currently involve biological replicates, so we discuss statistical approaches for differential expression analysis when replicates are and are not available.

## Designing sRNA sequencing experiments

### Sequencing technologies and inherent biases

The first decision to make when designing a sequencing experiment is which sequencing technology to use. Today there are two main varieties of next-generation sequencing: (1) sequencing by synthesis (SBS), employed by 454 sequencing http://www.454.com/; 454 Life Sciences/Roche, Branford, CT, USA) [77], Illumina (formerly called Solexa sequencing; http://www.illumina.com/; San Diego, CA, USA) [13], Helicos http://www.helicosbio.com/; Helicos Biosciences Corp., Cambridge, MA, USA) [78,79] and the latest entrant into the market, small-molecule, real-time sequencing, or SMRT, sequencing introduced by Pacific BioSciences http://pacificbiosciences.com/; Menlo Park, CA, USA) [80]; and (2) sequencing by ligation (SBL), used in SOLiD (Sequencing by Oligonucleotide Ligation and Detection; http://www.appliedbiosystems.com/; Applied Biosystems, Carlsbad, California, USA) [81] and Polonator sequencing http://www.polonator.org/; Dover Systems, Salem, New Hampshire, USA) [82]. Table 1 shows the current efficiency statistics for each of these methods as provided by the product websites, but the sequencing depth, speed and accuracy of these technologies are constantly increasing. Most of these approaches can be implemented as paired-end runs, in which both ends of each clone are sequenced, increasing the amount of information gleaned per fragment, but single-end runs are sufficient for the short length of sRNA [83-85].

**Table 1:** Comparison of next-generation sequencing technologies^a^

Technology	Approach	Approximate sequencing depth	Read length, nt	Paired ends	Accuracy	Individual molecule sequencing	Optimal for sRNA
Illumina (Solexa)	Synthesis	500 M reads/flow cell, 12 Gb/35-nt run	35 to 75	Optional	≥98% to 99%	No	Yes
454	Synthesis	1.6 M reads/flow cell or 500 Mb/run	400	Optional	≥99%	No	No
Helicos	Synthesis	300 to 500 M reads/flow cell	25 to 55	Optional	> 99.995%	Yes	Yes
SMRT	Synthesis	75 K reads/flow cell	1,000	N/A	99.30%	Yes	No
SOLiD	Ligation	2.4 B reads/flow cell or 300 Gb/run	35 to 75	Optional	≥99.94%	No	Yes
Polonator	Ligation	64 to 80 M mappable reads or 2.2.5 Gb/flow cell	13	Mandatory	98%	No	No

^a^nt, nucleotides; sRNA, small RNA; SMRT, small-molecule, real-time sequencing; SOLid, sequencing by oligonucleotide ligation and detection.

The choice of sequencing method often comes down to cost, read length and sequencing depth. Because sRNA are in the range of approximately 18 to 30 nt and high sequencing depth is necessary to observe rare species, Illumina and SOLiD are currently the most appropriate methods for sRNA sequencing studies (Table 1). Illumina uses a four-color, reversible terminator sequencing-by-synthesis technology to sequence one base at a time. SOLiD uses 16 dinucleotide probes, each labeled with one of four fluorophores, to sequence by ligation two nucleotides of each clone at a time. This means that four dinucleotide pairs share the same label, making the analysis of SOLiD data a little more complicated. An algorithm generates the nucleotide sequence of a particular base *n *from this color space by examining the labels for the overlapping dinucleotides *n *- 1, *n *and *n*, *n *+ 1 [81]. In this fashion, two different probes interrogate each base, which accounts for the reportedly high accuracy of this method. A single color call error, however, invalidates the sequence determination for all positions after this point. The read length and sequencing depth of Helicos sequencing make Helicos appropriate for sRNA sequencing as well, but this application has not been widely commercialized. For Helicos sequencing, cDNA molecules are polyadenylated and then annealed to immobilized oligo(dT) primers. Individual molecules are sequenced by sequential addition of each of the four nucleotides. One advantage of the Helicos method is that it allows for the sequencing of individual DNA molecules, eliminating the need for polymerase chain reaction (PCR) amplification and its inherent error rate. While Polonator sequencing allows for 26-nt reads at great sequencing depths, a 3- to 4-nt sequence gap remains in the middle of each read, which is not ideal for sRNA experiments.

### Library preparation and inherent biases

Recent data have shown that the library preparation method, more than the sequencing technology, can significantly affect the diversity and abundance of the sRNA that are sequenced [86]. For differential expression analyses comparing the relative abundance of the same sequence in different libraries, this is not a problem because all libraries will be affected equally by biases due to library preparation. Despite the digital nature of sequencing data, however, the relative levels of different sequences within the same library will be affected by these biases. Some sequences present in the biological samples may even be absent in the libraries because of preparation bias.

Illumina and SOLiD sRNA sequencing libraries are made by ligating RNA adapters of known sequence to the 5' and 3' ends of single molecules in a purified sRNA population. Alternatively, SOLiD sequencing can be performed by *in vitro *polyadenylation of the 3' end of the sRNA and addition of a 5' adapter [86]. In either case, the adapter-ligated sequences are reverse-transcribed, amplified by PCR to increase the size of the library, applied to the platform and amplified again *in situ *to form millions of clusters of DNA of the same sequence. Then these clusters are sequenced in parallel.

Three steps in this process have the potential to influence the sequencing results: adapter ligation, reverse transcription and PCR amplification. Adapter ligation is the most important. The adapters have typically been ligated to each sRNA using T4 RNA ligase 1, which is capable of ligating two single-stranded oligoribonucleotides, where the acceptor nucleotide (≥3 nt long) has a free 3'-hydroxyl group and the donor (≥1 nt) has a 5'-monophosphate [87]. The ligation efficiency of T4 RNA ligase 1 is very sensitive to nucleotide base composition at the ligation site and to sRNA modifications, however, and not all sRNA can act as donor substrates for the enzyme. Studies have suggested that the sequences of both the acceptor and the donor have an effect on ligation efficiency [86-91], but the acceptor sequence is more important [87]. The identity of at least the three 3'-most nucleotides of the acceptor affects ligation efficiency [87,91], with a different base preference at each position (5'-nucleotide: A > G ≈ C > U; middle nucleotide: A > C > U > G; 3'-nucleotide: A > C > G > U when using a pUUUCp donor) [91]. The donor sequence appears to be less important, but the bias for the 5' nucleotide is C > U ≥ A > G [88,89].

Many sRNA are modified, and these modifications can also make them poor substrates for T4 RNA ligase 1. In particular, miRNA, siRNA, hc-siRNA, ta-siRNA and nat-siRNA in plants, siRNA and piRNA in insects and piRNA in animals are known to be 2'-*O*-methylated on the 3' end by the conserved methyltransferase HUA ENHANCER 1 (HEN1) (reviewed in [92]), and this modification lowers ligation efficiency by T4 RNA ligase 1 by 30% to 72%, depending on assay conditions [93-95]. The 2'-*O*-methylation also introduces a sequence bias for the 3' nucleotide of the acceptor at the ligation site, such that the efficiency is G = C > A > U [95]. Unlike previous studies, the study by Munafó *et al*. [95] did not find sequence bias at the acceptor site in unmethylated sRNA. Both of these issues are eliminated by using a truncated version of a closely related ligase, T4 RNA ligase 2, with a preadenylated 3'-RNA adapter [95], so this enzyme is being used more and more for library preparation. Illumina's first-generation sRNA library preparation kits used T4 RNA ligase 1 for the ligation of both the 5'- and 3'-adapters, but their Small RNA version 1.5 and TrueSeq™ RNA Sample Preparation kits use the truncated form of T4 RNA ligase 2 for the ligation of the 3'-adapter. T4 RNA ligase 1 is still required for the ligation of the 5'-adapter, however, because of the need by the truncated T4 RNA ligase 2 for a preadenylated donor, which in this case is the sample itself. Thus, sequence bias is eliminated in only one of the two ligation reactions. To test whether an sRNA is 3'-modified or to specifically clone 3'-modified products, sRNA can be oxidized with NaIO_4 _followed by β-eliminated at an alkaline pH. This treatment removes the 3'-most nucleotide from all sequences with 2',3'-OH groups (that is, unmodified sRNA), but not from modified sRNA, leaving a 3'-phosphate [96-98], which is not a substrate for T4 RNA ligase 1 or 2.

Because T4 RNA ligase 1 requires a 5'-monophosphate on the donor sequence, sRNA lacking this group are absent from standard libraries. A large population of 5'-ligation-resistant secondary siRNA was found in *C. elegans *[51,52]. These secondary siRNA are involved in the perpetuation of RNA interference (RNAi) and have a 5'-triphosphate, which is not a substrate for T4 RNA ligase 1. sRNA with 5'-diphosphate or 5'-triphosphate have also been found in the single-celled eukaryote *Entamoeba histolytica *[99]. The 5'-caps similarly block ligation by the enzyme and have been seen on 18- to 25-nt sRNA associated with the human hepatitis delta virus and on some RNA under 200 nt in human cells [100,101]. Both of these ligase-resistant 5'-modifications can be removed by pretreatment with tobacco acid pyrophosphatase before ligation of a 5'-adapter [101]. Alternatively, a 5'-adapter-independent method can be used [51,99,100]; however, this approach is not compatible with Illumina and SOLiD sequencing technologies. The importance of considering such a method, however, is highlighted by a study by Pak *et al*. [51], who studied RNAi-induced *C. elegans *that used a 5'-adapter-independent library preparation protocol. In contrast to work that did not account for the possibility of 5'-ligation-resistant sRNA, which suggested that miRNA vastly outnumbered siRNA, they demonstrated that the two classes are actually found in similar degrees of abundance [51].

Because sRNA acts as the donor during the 5'-adapter ligation and as the acceptor during the 3'-adapter ligation, the best solution for avoiding this bias would be to use a ligation-independent library preparation. Such a method has been applied to the generation of Illumina sequencing libraries [10] and would be applicable to SOLiD sequencing as well. This method involves using *Escherichia coli *poly(A) polymerase (PAP) to polyadenylate the RNA molecules and then performing a reverse transcription reaction with an oligo(dT) primer having both 5'- and 3'-adapter sequences at the 5' end of the primer. The products are then circularized and cut with a restriction enzyme that cleaves between the 5'- and 3'-adapters, yielding the typical linear read of 5'-adapter, clone and 3'-adapter. Ligation-independent methods that rely on 3'-polyadenylation of the sRNA population, such as this technique and the one used for Helicos sequencing, may be better than ligation-dependent methods, but they are still not perfect. PAP has a bias for the 3'-nucleotide A = G > C > U, but the efficiencies of the different bases are within twofold of each other [95]. As seen with T4 RNA ligase 1, 2'-*O*-methylation greatly reduces the efficiency of PAP by up to 10-fold, with the sequence bias altered to 2'-*O*-meG > 2'-*O*-meA = 2'-*O*-meU > 2'-*O*-meC [93-95].

While adapter ligation is probably the largest potential source of bias, bias can also be introduced during reverse transcription and amplification. The 2'-*O*-methylation of sRNA reduces the efficiency of reverse transcription as well as adapter ligation [95,102]. The step of PCR amplification during library preparation can be a problem with sequences that have very low or very high guanine-cytosine (GC) content, reducing the likelihood that these sequences will be represented in the final population. Two techniques that do not require the initial library amplification and are compatible with Illumina sequencing have been used for DNA-seq and RNA-seq, and both methods provide a less biased library preparation for low GC sequences [103,104]. These approaches remain to be tried with sRNA libraries and still require the standard amplification within the Illumina flow cell to generate clusters of identical sequences. The Helicos system will provide a truly amplification-independent sequencing protocol because it does not require PCR in the library preparation and sequences only single molecules, not clusters of molecules.

### Multiplexing

High-throughput sequencing can be costly when loading only one sample per sequencing lane. To help improve cost efficiency, users can multiplex two or more samples in a single lane using bar coding [105-113]. As the number of reads per run has increased (Table 1), sufficiently deep sequencing can be achieved even when running multiple samples in the same lane, with the number of multiplexed samples depending on the desired depth. Multiplexing either incorporates a unique sequence called a bar code into the 5'- or 3'-adapter of each library to be run in the same lane or adds the bar code during a PCR step after adapter ligation, an approach that minimizes ligation bias. All of the reads in a lane can be sorted into their respective libraries using their bar codes after sequencing has taken place. Because of the inherent error rate of sequencing, it is recommended that bar codes be long enough so that each pair varies by multiple substitutions, thereby reducing the likelihood that sequencing errors in the bar code will result in assigning reads to the wrong sample [107,112]. In particular, Illumina sequencing has a tendency to erroneously incorporate adenine more than the other bases [114], which should also be taken into account when designing your own bar codes. Multiplexing library preparation kits are now available for both Illumina and SOLiD. In both cases, the bar code is located within one of the adapters and separated by multiple bases from the ligation site, reducing the likelihood that the bar code will introduce any ligation bias. Helicos is also compatible with bar coding, though it requires a ligation step not in the original protocol. The one downside of using a bar code is that it may reduce the maximum length of the sRNA that can be sequenced, trimmed and assigned to a sample. However, the latest multiplexing systems for the Illumina and SOLiD machines incorporate the index into the 3' PCR primer and perform a second reaction specifically to sequence the bar code. This type of approach has numerous advantages, such as reducing or eliminating ligation bias, ensuring long reads across the sRNA and enabling multiplexing that reduces sequencing costs.

### Replication

Several reports have used technical replicates, that is, the same library sequenced multiple times or independent libraries constructed from the same biological sample, to demonstrate the high reliability of Illumina [86,115-118] and SOLiD sequencing [86]. Similar results are possible for biological replicates [115,118,119]. Because of the high cost of deep sequencing, most experiments published to date have not used biological replicates, even though they can increase the statistical significance and reduce both false-positive and false-negative rates. With biological replicates, the significance analysis of microarrays (SAM) [115] and the Bioconductor program edgeR [118,120] can be applied to differential expression analysis of sequencing data, as we discuss later in the section "Differential expression analysis". Standards for deep sequencing experiments remain to be agreed upon, but as sequencing costs go down, sequencing depths further increase and multiplexing becomes more widely adopted, the requirement for biological replicates in differential expression experiments will surely follow.

## Preprocessing of sequencing data

**Figure 2:**
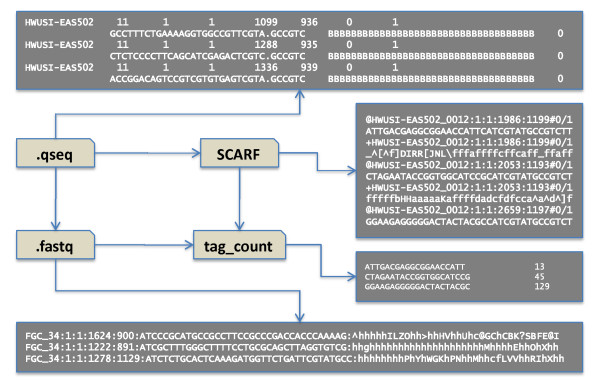
**Sample file formats for small RNA libraries**. Illumina machines generate .bcl files, which are in binary form and are not human-readable. These files are converted into .qseq files, which record the most likely sequence and a quality score for each read. Scripts are available to convert files in .qseq format into .fastq or SCARF format (Solexa Compact ASCII Read Format). Files in these formats are often converted to a "tag count" format so that they can be easily stored and analyzed.

The raw data of a sequencing experiment typically comprise a series of image files: one image per cycle of nucleotide addition for Illumina or dinucleotide ligation for SOLiD. Because of the size of flow cells, each one is subdivided into a number of "tiles" for imaging purposes. Thus, there is a series of images for every nucleotide. The images contain thousands of spots, one spot for every cluster, with a cluster representing one read. Each of these files must be analyzed to designate one of the four nucleotide bases (Illumina) or color space call (SOLiD) for each spot on the image, and then the data from each image for the same spot must be combined to give full sequence reads, one per spot. Each technology has its own specifications regarding the file formats used; for example, Illumina recently changed its standard output format from .qseq, which uses ASCII-64 encoding of Phred quality scores (a widely accepted metric to characterize the quality of DNA sequences), to .bcl, a binary format containing base call and quality for each tile in each cycle. SOLiD systems use .csfasta to encode color space calls and .qual files to record the quality values for each sequence call. Because one color call error will affect the sequence of all 3'-nucleotdies, SOLiD data are maintained in color space for much of the preprocessing. Figure 2 demonstrates a sample pipeline for Illumina data files.

For many sequenced reads, ambiguous bases will exist. Ambiguous bases are the result of low confidence in any particular nucleotide. In the case of Illumina, a probability is assigned for a given nucleotide being each of the four bases. For a sequence designation to be assigned, the likelihood of the most likely base has to be at least 1.5 times greater than that of the next highest base; otherwise, the position in question will be deemed an ambiguous base. Different sequencing platforms and/or software pipelines have alternative approaches for handling ambiguous reads, usually denoted with an "N" in a sequence. Some will simply discard any sequence with an ambiguous read if the sequencing depth is sufficient, while others will assign the most likely base call at that nucleotide in an attempt to maximize the number of reads. A very sophisticated approach to this step is to record each read as more than a static sequence by using a probability matrix to record the probability of each nucleotide at each position [12]. This additional information can help recover reads that would otherwise be classified as sequencing errors. For example, the most likely sequence for an ambiguous read, according to its probability matrix, might not map to any genomic locus, but the second most likely sequence might correspond to a known miRNA. This approach is likely to increase the number of usable reads for any given library, but it is undecided whether this increase is enough to warrant the increase in computational complexity that it brings. This approach will also likely mistakenly assign the sequence of some reads. The location of the ambiguities may also allow some reads to be saved. Ambiguities in the middle of a read will require that it be discarded from further analysis, but if it is within the adapter sequences, the read may still be retained.

The next step in processing next-generation sequencing data is to trim or remove any adapter sequences. Because these adapters are artificially introduced and are not part of the organism's transcriptome, it is necessary to remove any remnants of them before attempting to map the libraries against a reference genome. Trimming scripts require a minimum number of bases for adaptor recognition, so the maximum usable read length of Illumina and SOLiD is less than the total number of sequenced bases. This also means that longer sRNA may be lost as a result of an insufficient adapter sequence for matching and trimming. This is not a problem for the typical 19- to 30-nt sRNA, as current technologies generate sequences > 36 nt. The process of removing adapters can be inefficient because it is possible (even likely) that sRNA sequences contain subsequences of the adapter. Thus, researchers must be careful when defining exact rules for determining which sequences to keep, which ones to trim and which ones to throw out altogether.

The final steps before data analysis can begin are to count the abundance for each distinct tag in a library and to map distinct tags to a reference genome if one exists. Calculating the abundance is computationally trivial, given current sequencing depth and standard computational limitations, so many researchers use their own programs for this step. Genome mapping, on the other hand, can be computationally expensive, but fortunately there are a number of publicly available programs to perform this task, such as SOAP [121] and Bowtie [122], each with its own benefits and limitations. Some programs use multithreading and efficient memory allocation to maximize mapping speed.

The number of trimmed reads in a given library that will align perfectly to a reference genome depends on issues specific to the organism, the sample or the sequencing run, as well as on decisions made during data analysis. The completeness of the genome sequence is a major factor. Even in so-called "complete" genomes, there are highly repetitive regions (such as in centromeres and telomeres) that remain undetermined. Because a large number of sRNA originate from these locations, many reads will incorrectly fail to map to the genome. The sequence divergence between the reference genome and the sample will also have an effect. Low-quality sequencing runs will have reads riddled with erroneous base callings, causing them to be classified as nongenomic as well.

**Figure 3:**
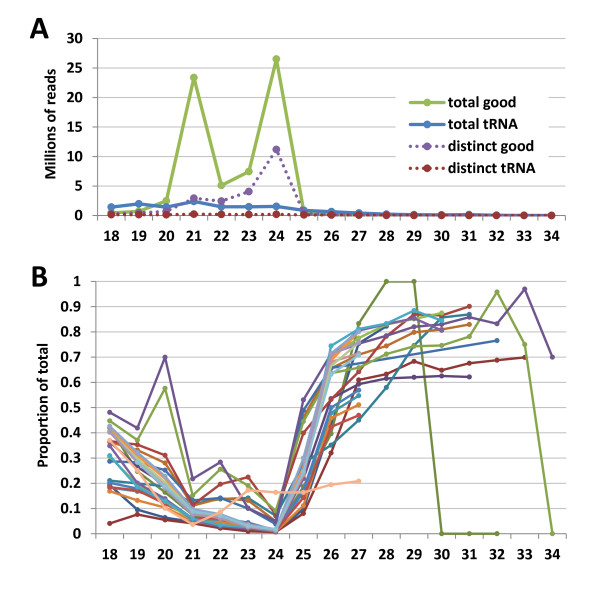
**Small RNA (sRNA) reads derived from structural RNA versus other sRNA-generated loci**. **(A) **The number of total and distinct reads for all genomic sequences divided into those derived from ribosomal RNA, transfer RNA (tRNA), small nuclear RNA (snRNA) or other "structural" noncoding RNA-derived and other categories for each size class from 18 to 34 nt across 51 publicly available *Arabidopsis *sRNA libraries. We typically refer to the sRNA from nonstructural loci as "good" sRNA. **(B) **The percentage of tRNA-derived reads for each size class from 18 to 34 nt across 24 publicly available wild-type *Arabidopsis *libraries. Because of variations in sequencing read lengths among libraries, some libraries are missing data for sizes above 27 nt or 31 nt.

There are also some data analysis decisions that will influence the number of reads that align to a genome, including minimum read length, how to handle reads mapping to multiple genomic loci and how many mismatches to allow. Shorter sequences are more likely to map to multiple loci in the genome. Because sRNA researchers are generally interested in Dicer-mediated cleavage events, and because the shortest known Dicer products are 19 nt in length, it is recommended that any reads shorter than 18 nt be excluded. In plants, because the dominant size classes are miRNA and hc-siRNA, with the bulk of these being 20 or 21 nt and 23 or 24 nt, respectively, the data should demonstrate a significant decrease in the number of both distinct and total 18- or 19-nt and > 25-nt reads. Figure 3 demonstrates how reads shorter than 20 nt or longer than 24 nt are mostly derived from tRNA, rRNA, small nuclear RNA (snRNA) or small nucleolar RNA (snoRNA) loci.

**Figure 4:**
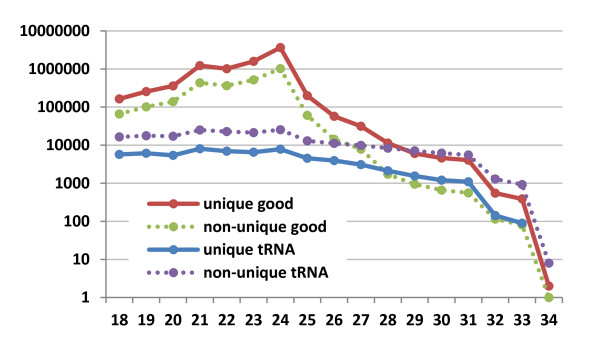
**Repetitiveness of small RNA (sRNA) reads measured across sizes**. The number of total reads for all uniquely and nonuniquely mapping genomic sequences divided into ribosomal RNA- or transfer RNA-derived and other (also known as "good") categories for each size class from 18 to 34 nt across 51 publicly available *Arabidopsis *sRNA libraries. For each size class, structural RNA-derived reads are more likely to map nonuniquely mapping genomic sequences (that is, to more than one genomic location), whereas good reads are more likely to map uniquely mapping genomic sequences (that is, to one genomic location).

Several strategies have been employed to handle reads that map to multiple loci, also known as multireads. Reads that map to only one locus are called unique reads, which should not be confused with the distinct reads, which are reads with different nucleotide sequences. Figure 4 shows the relative abundance of unique and nonunique reads across all sRNA size classes. In some cases, researchers have chosen to exclude all multireads from analysis [123], or to exclude those multireads mapping to more loci than some threshold [124,125], as many of these will map to centromeres and telomeres. However, this will result in a loss of sequencing depth. When choosing to keep multireads, the problem arises how to allocate those reads between the different possible source loci. The two most common approaches are to allocate the total number of copies of a read to each mapped locus or to divide the number of copies evenly among the mapped loci. Allocating all copies to each locus ignores the fact that this is biologically impossible, but allows for the possibility that any locus might be the sole transcriptional source of a read. Distributing the copies evenly, while reflecting a biologically possible scenario, precludes such a possibility. A more sophisticated approach is to estimate the proportion of multiread transcriptions at each locus by examining the levels of uniquely mapping reads at nearby loci [126,127]. This approach has several names, but we shall refer to it as "probability mapping," since it involves estimating the probability that a transcript originated from each associated locus. The basic idea of probability mapping can be explained with this simple scenario. Suppose a multiread maps to genomic loci L1 and L2 and that the number of uniquely mapping reads overlapping L1 greatly outnumber those that overlap L2. Intuitively, we can presume that most of the copies of the multiread in question originated from L1, since there is likely a higher level of transcription occurring at L1 than at L2. The proportion of copies allocated to L1 is then approximately equal to the proportion of uniquely mapping reads overlapping L1 compared to those at L2. While it remains unknown whether the presence of uniquely mapping reads is an indication of a higher overall level of transcription, the data from applications of this technique seem to support the idea.

The number of mismatches to allow when performing genome mapping can be a difficult issue to resolve. Individual specific DNA polymorphisms and posttranscriptional sequence modifications, which have been seen in RNA from mitochondrial and plastid genomes, tRNA and miRNA, will also cause some reads not to map to the genome. Computational techniques that allow indels and mismatches when performing genome mapping are capable of "recovering" these modified reads that would otherwise be classified as nongenomic [125,128,129]. Allowing mismatches increases the number of raw reads that will map to the genome but also decreases the likelihood that those reads originated from the matched loci. Because of the short length of sRNA, it is generally recommended that only perfectly matched reads be utilized, unless specific known polymorphisms or posttranscriptional RNA sequence modifications exist between the reference genome and the sample in question.

### Quality control

Once sRNA data have been preprocessed, it is common for researchers to verify the quality of the data before moving on to normalization and analysis. There are several ways to perform quality control on sRNA data. Each base of every Illumina sequenced read or each color call of every SOLiD sequenced read is given a quality score, which can be used to calculate an average error rate for each cycle of a sequencing run. While it is normal for the error rate to increase toward the end of a run, for a good run the average error rate throughout should be relatively similar and close to the expected rate for the technology. Creating size distribution graphs should reveal peaks of sequences corresponding to the dominant size classes. For example, in *Arabidopsis*, the dominant classes are 20 or 21 nt and 23 or 24 nt, which correspond to miRNA and hc-siRNA, respectively. Libraries made from high-quality RNA should have low levels of sRNA corresponding to highly abundant mRNA. Libraries made from green tissues of plants, for instance, should have low levels of sRNA for genes encoding the highly expressed photosynthetic proteins. Computing the levels of other RNA types, such as tRNA or rRNA, among different libraries in a data set may or may not be informative, as the relative level of tRNA can vary significantly. For example, from 51 public *Arabidopsis *sRNA libraries in our databases, tRNA represented from 4% to 40% of the total number of sequenced reads. Ideally, the level of nongenomic reads should also be similar between libraries to be compared.

## Data normalization

### Lessons from microarrays

The more than 20-year history of microarray experiments provides a good starting point for considering how to normalize next-generation sequencing data. While there are many technology-specific issues involved when handling raw microarray and sequencing data, the basic problem is still the same: how to convert raw data, in the form of image files, to numerical data, such that any expression differences between samples are due solely to biological variation, not to technical, experimentally introduced variation. In the case of microarrays, technical bias can be introduced during sample preparation (differences in RNA isolation, quality and amplification; target labeling; total amount of target; dye biases for spotted arrays; and so on), array manufacture (array surface chemistry, sequences used for the probes, locations of the probes within a gene, array printing for spotted arrays, scratches and so on) and array processing (hybridization conditions and scanning intensity and settings). Failing to properly remove these biases can lead to false conclusions when making comparisons within a single array or between two different arrays. Normalization attempts to remove technical bias without introducing noise.

Normalization requires two basic decisions: (1) which subset of genes (also called the normalization baseline or reference population) to use to determine the normalization factor and (2) which normalization method to employ [130]. These two choices are independent, such that a given reference population can be used in combination with any of the different normalization methods. A good reference population is invariant in expression, meaning that the true expression levels are constant across biological treatments and span the entire expression range. Reference populations that have been used previously for microarray normalization include housekeeping genes [131], spike-ins of nonendogenous RNA or genomic DNA, an algorithmically identified set of invariant genes [29,132-135] and all genes [130]. Housekeeping genes are typically used for normalizing northern blot analysis results and quantitative reverse transcription PCR (qRT-PCR) because of their supposedly constant expression level, but it has become ever more apparent that even these genes can vary in their expression [136-141]. Commercial arrays typically have probes for nonendogenous genes, and *in vitro *transcribed RNA from these genes can be used as spike-ins at various steps in the target preparation and array hybridization procedure. The point chosen will determine how much and what kind of technical variation will be corrected by the normalization. Genomic DNA has also been used for normalization because the concentration of a control sequence is readily known. In the absence of knowledge regarding invariant genes, algorithms have been developed that identify a set of invariant genes from the set of arrays themselves. These genes are discovered by comparing expression-ranked lists of all of the probes in each array to find the most rank-invariant genes [29,132-135]. This method is advantageous because it makes no assumptions about the expression patterns of individual genes. Normalization is generally improved by increasing the size of the reference population, which has been a disadvantage of spike-ins because only a few sequences are typically added. As an alternative to using a subset of probes for normalization, all probes can be used. This type of normalization assumes that because the RNA content is constant between treatments and most of the genes do not change in expression between treatments, the median or mean expression across all of the genes is unchanged.

There are many different algorithms for normalizing microarray data on the basis of the chosen reference population, but they fall into four main categories: linear scaling (as in the MAS5.0 algorithm), nonlinear scaling (as in locally weighted linear regression (LOWESS), cyclic LOWESS), quantile normalization (as in robust multi-array average (RMA), GC-RMA (a variation of RMA), dChip) and variance stabilization normalization (VSN), two of which (linear and nonlinear scaling) have been applied to sRNA sequencing data as we will see later in the section "Normalization methods". Linear scaling uses the reference population to determine a single factor by which the population varies when compared to a set target, such as a predetermined mean or median expression value. The expression of each probe or gene on the array is multiplied by this factor to achieve the normalized expression value. The advantage of using linear scaling is that the scaling factor is determined independently for each sample, unlike the other approaches, which normalize the data with reference to the other arrays in the data set. Linear normalization of microarray data has been largely abandoned, though, because expression values are not necessarily linear, particularly at the extremes [142]. In attempt to overcome this problem, nonlinear scaling methods have been developed that, for a given pair of arrays or for an individual array and the mean or median data derived from all of the arrays in question, first fit a curve to the expression values of the reference using LOWESS or splines and then normalize the data such that the average fold change when comparing any two arrays is 1 (that is, no change) across the expression range. Thus, a scaling factor is determined independently for small windows across the entire expression range. Quantile normalization uses a nonscaling approach that assumes that most genes are not differentially expressed and that the true expression distribution is similar between different samples [142]. The average distribution of the reference population is determined from all of the arrays in question, and then each array is normalized to have this same distribution. Variance stabilization normalization likewise assumes that most genes are not differentially expressed. Using a generalized logarithmic transformation, VSN methods fit the data such that the variance is equal across the expression range, allowing for greater precision for low expression values, which are generally subject to greater variance [143-145]. Many studies have been performed comparing these different normalization methods, but beyond the opinion that linear scaling is not as ideal because of the analog nature of microarray data, the general conclusion is that there is no single "best" normalization method [31,131,142,146-149]. Even though the data are digital, the same is likely to be true in the case of RNA sequencing experiments as discussed below in the section "Normalization methods".

### Sources of nonbiological variation in sRNA sequencing experiments

There are a number of nonbiological sources of variation that can add noise to sRNA sequencing experiments. RNA quality is a major issue because low quality can result in an increase in sequencing of degradation products. As discussed above in the section "Library preparation and inherent biases", the choice of library preparation methods has a significant impact on the makeup of the library because of biases in ligation, reverse transcription, PCR amplification or polyadenylation efficiency. While not currently done, it may be possible to develop methods to correct for these biases. One issue that can be dealt with to some extent by normalization is differences in sequencing depth between libraries. More total reads equate to a higher likelihood of any particular sequence's appearing in a library, and standardizing the total number of reads per library or sequence run is not a realizable goal. One way to reduce the impact of this kind of variation (or other technical variations encountered as a result of the sequencing procedure itself) is to sequence all of the libraries to be compared at the same time or to use multiplexing to run the samples in the same lane or at least on the same flow cell.

Microarray and sequencing experiments start with equal amounts of total RNA when constructing a library or a labeled target. When performing differential expression analyses using such data, an inherent assumption is that a set amount of starting RNA comes from the same number of cells in each sample. It is well known, though, that transcription rates change depending on the stage of growth, development or environment of the cell, tissue, organ or organism. Thus, this assumption can result in over- or underestimation of differences between samples. This issue is probably most significant when comparing different stages of growth or development. Studies of the per-cell abundance of sRNA in different experimental conditions have not been performed, but such studies might help improve our estimates of differential expression as well as our knowledge of the biology of sRNA.

### Selecting a normalization baseline for sRNA sequencing experiments

Three reference populations for normalization have been used with sRNA sequencing experiments: spike-ins, all "good reads" and all reads. As discussed earlier in the section "Lessons from microarrays", housekeeping genes have been shown to be nonideal for normalizing microarray data because of their variable expression [136-141]. In the case of sRNA, few "housekeeping" sequences have been delineated. The identification of rank-invariant sRNA sequences would help to establish a statistically significant baseline for normalization, but this has not been done to date. RNA spike-ins of foreign sequences have proven useful, however, to account for multiple sources of variation in sequencing experiments, particularly when the spike-in RNA have been added to the total sample RNA prior to library preparation [115]. Fahlgren *et al*. [115] added multiple spike-ins at different concentrations to cover a range of abundances. Some sequences were more likely sequenced than others even when added at the same concentration, possibly as a result of sequence biases, so it is probably best to include multiple spike-ins of varying base compositions for each of the concentrations to be tested. Spike-ins also have proven useful in demonstrating the accuracy of some downstream data analyses [126,150].

Many other studies have used all reads or, more often, all "good reads" for the normalization baseline, which is comparable to using all probe sets when normalizing microarrays. Good reads are defined as all tags that map to a reference genome, except those associated with tRNA, rRNA, snRNA, snoRNA or other structural RNA [124,151]. This approach helps to mitigate the effects of bad sequencing runs and contamination with foreign RNA, both of which result in higher numbers of sequences that do not map to the reference genome. Experiments focusing on a specific RNA type, such as miRNA, may choose to use only these sequences for the normalization baseline [152,153].

In sRNA sequencing experiments, the majority of distinct reads will be sequenced in only one copy and often will be observed in only a single library. Because these sequences can act as outliers, it is sometimes best to eliminate them from the normalization baseline as discussed in the next section.

### Normalization methods

Once a normalization baseline has been chosen, there is still the decision which normalization method to use. Existing methods can be classified as either linear or nonlinear. Linear total count scaling is perhaps the simplest of all existing methods. It involves using the summation of all reads belonging to the normalization baseline as a "library size," choosing an appropriate "control" library size (either the actual size of a control library or the average size of all libraries in the experiment) and then multiplying the abundance of each individual read by the normalization value (control divided by library size). This method has been widely applied to different types of data, including sRNA Illumina data, mRNA Illumina data [154] and PARE Illumina data [151]. Linear total count scaling has been shown to be no better than the analog data of microarray experiments for detecting differentially expressed genes [154]. A slight variation of this method is to use the number of distinct sequences, rather than the total abundance, as the size of each library [155].

Total count scaling is computationally simple but, for some experiments, biologically naïve. Consider this hypothetical scenario in which total count scaling fails: If sample A contains all reads from sample B, as well as a novel set of reads equal in size to the first set, total count scaling will result in underrepresenting reads from sample A and overrepresenting reads from sample B [120]. Total count scaling is particularly inefficient in the context of sRNA sequencing because it ignores the number of distinct reads within each sample. One proposed method that incorporates this number is quantile-based normalization, which uses the upper quartile of expressed genes (after excluding genes not expressed in any library) as a linear scaling factor [154]. (Note that this differs from quantile normalization, which scales data within each quantile separately.) The quantile-based method has been shown to yield better concordance with qRT-PCR results (with a bias near zero) than linear total count scaling, making quantile-based normalization better at detecting differentially expressed genes [154]. This quantile-based method has been used with RNA-seq data, where all reads per gene have been grouped together to yield one total per gene, but it has not been used with sRNA sequencing data. Our attempts to apply this approach to sRNA sequencing data (about 0.5 to 2 million distinct reads per library) found that the 75th-percentile sRNA were found at only one or two copies per library. Even grouping sRNA by gene or by 500-bp sliding window found very low copy numbers at this percentile. As a result, this method may need further modification to be applied to sRNA data, such as not considering distinct reads sequenced only one time or raising the percentile used for the normalization.

Even quantile-based normalization has its limitations, because it assumes a similar distribution of abundances per distinct read among all libraries being normalized. It is not yet known how accurate next-generation sequencing is with regard to read distribution. It is possible, however, to properly normalize libraries that may not have similar abundance distributions by using linear regression [123]. This method involves performing linear regression by comparing the abundance of each baseline element between two samples or between one sample and the mean or median of all samples, and then using the slope of the regression line as a linear scaling factor.

Because the total RNA output of each sample is unknown, linear total count scaling and other naïve methods can lead to underrepresentation of counts from high-output samples. Highly expressed genes (or other genomic elements) can sometimes take up too much "sequencing real estate" in a sample. The number of reads that map to a particular gene depends not only on gene length and expression level but also on the composition of the RNA population being sampled [120]. In some studies, it is assumed that most genes are not differentially expressed and thus that their true relative expression levels should be pretty similar. The trimmed mean of M value (TMM) normalization method exploits this fact by calculating, for each baseline element, the log expression ratio (M values) of the experimental sample to a control sample (or the mean or median of all samples) and using their trimmed mean as a linear scaling factor. Although Robinson and Oshlack [120] applied this method to genes using RNA-seq data, it could be applied to individual sRNA sequence counts as well.

**Figure 5:**
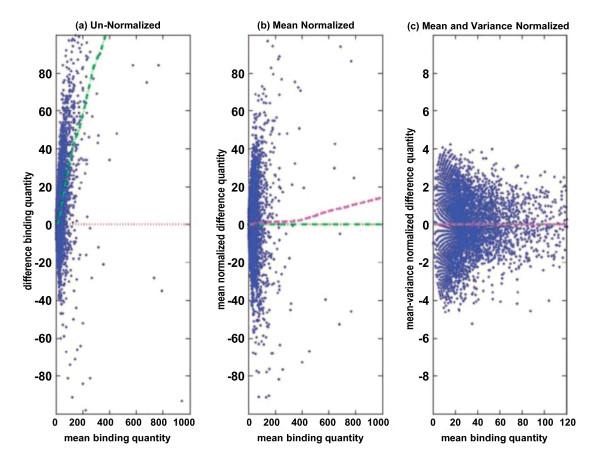
**Example of two-step nonlinear normalization**. An example of the normalization process applied to the binding quantity difference regarding breast cancer data on human chromosome 1 between (1) MCF-7 control and (2) MCF-7 with E2 stimulation. **(A) **Raw data with clear bias toward the positive direction. **(B) **Data normalized with respect to the mean. **(C) **Data normalized with respect to both mean and variance (*x*-axis is zoomed in). Green dashed-dotted line and magenta dashed line represent the locally weighted linear regression line with respect to the mean and variance, respectively. Red dotted line represents the zero difference line. Reproduced with permission from Oxford University Press from Taslim *et al*. [156].

All of the normalization methods discussed thus far are linear scaling methods, and they suffer from an inherent flaw in assuming that the level of noise in an sRNA library is directly proportional to the size of the library. A two-step nonlinear regression method can be used to eliminate nonlinear noise without making any assumptions about its shape [156]. A previously published implementation of this method is shown in Figure 5. This method uses the number of sequences mapping to each genomic window as well as the averages of these counts across the set of libraries. While this particular normalization method assumes that the data include only uniquely mapping sequences, multireads could be included by using probability mapping (described above in the section "Preprocessing of sRNA data") to estimate the total number of transcripts originating from within each genomic window. The first step is to regress observed counts of differences (control minus sample) on the mean to estimate fitted values and then subtract these fitted values from the observed difference counts. This results in each observed count's being transformed into a mean normalized difference. The second step is to estimate the moving mean absolute deviation (by regressing the absolute value of mean normalized differences on absolute mean counts) and then divide the mean normalized difference counts by the estimated mean of absolute deviation.

A summary of the normalization methods discussed here is given in Table 2. Because modern computational standards make most of the more advanced normalization methods relatively trivial, especially when compared to the task of genome mapping, we recommend that researchers not hesitate to use the more sophisticated approaches described herein. In particular, the methods implemented by Robinson *et al*. [120] (TMM) and Taslim *et al*. [156] (two-step nonlinear regression) seem to account for many flaws inherent in total count linear scaling, which has been the predominant normalization method of choice. A study comparing these two methods, as well as others, with each other would help to provide a much-needed "gold standard" for normalizing sRNA data. We also recommend using absolute counts, rather than log ratios, when performing normalization, as log ratios fail to account for the vast differences in magnitude evident in many sRNA data sets but absent from microarray experiments.

**Table 2:** Comparison of sRNA normalization methods^a^

Method	Computational complexity	Control required	Units normalized
Total count scaling	Low	No	Reads
Quantile-based scaling	Medium	No	Reads
TMM	High	Yes	Reads
Linear regression	High	Yes	Reads
Nonlinear regression	Very high	Yes	Genomic windows

^a^sRNA, small RNA; TMM, trimmed mean of M value.

## Differential expression analysis

Once sRNA libraries have been normalized, there are many different analyses that can be performed on them, but most fall under some category of differential expression analysis. Differential expression analysis can be performed on (1) individual sequences of interest, such as miRNA; (2) genomic elements, such as genes or transposons; or (3) discrete sRNA-generating genomic loci, also known as "clusters" or "bins." Clustering or binning involves dividing the genome into windows of equal size and summing all normalized counts for tags mapping each window. For experiments involving sRNA data, clustering is not ideal when comparing genomic elements with specific, singular mature sequences, such as miRNA, but can be useful in identifying differentially expressed regions in promoters, noncoding DNA or previously unannotated genes.

The methods for identifying genes expressed differentially with statistical significance differ depending on whether biological replicates were performed. The approach to identifying differential expression between digital tag counts first implemented by Audic and Claverie [157] is particularly sensitive to small differences in low tag counts and is useful for comparing data sets without replicates. Their A-C statistic involves computing the probability that two independent digital measurements of a particular sequence (or set of sequences) come from similar populations. As the actual values being compared increase, the minimum fold change between them recognized as significant decreases. Although this approach relies upon a single measurement for establishing an assumed Poisson distribution for a given sequence, it has been shown that this assumed distribution is never far from the true (but unknown) Poisson distribution [158]. The original implementations by Audic and Claverie [157] were for relatively small data sets (< 10 K reads) and modern sRNA data sets are several orders of magnitude bigger, but the statistical principles guiding the approach remain the same. Thus, the A-C statistic has become popular among biologists seeking to perform comparisons between large RNA data sets [124,158-160]. There has been at least one study, however, that demonstrated a poor fit between RNA-seq data and a Poisson distribution [161]. The nature of these types of data makes it difficult to identify a "true" distribution, leaving researchers to assume a distribution that they see most fit. Other distributions assumed include binomial [123] and negative binomial [120]. It should also be noted that Audic and Claverie [157] provided an alternative formula that allows for both normalization and differential expression analysis, but this alternative formula is not recommended for normalization purposes as it essentially implements a total count linear scaling and does not exclude tRNA or nongenomic reads.

For differential expression analyses on data sets with replicates, at least two approaches have been implemented recently. Bioconductor http://bioconductor.org/ offers a software package called edgeR (empirical analysis of digital gene expression in R) that detects differentially expressed genes in a replicated experiment using an overdispersed Poisson model (a Poisson model allowing for greater variability) and an empirical Bayes procedure to moderate the degree of overdispersion [162]. By using a parameter to estimate the dispersion between replicates, the model can separate biological variation from technical variation. The edgeR program takes raw sequence counts and total library counts as input parameters, so the data do not have to be normalized first. This approach was used by Eveland *et al*. [118] to identify differentially expressed genes from maize RNA-seq libraries. Using qRT-PCR, significant differences were validated for 80% of genes identified as differentially expressed. Differential expression detection was possible on tags found in more than 10 copies, but the statistical strength increased with higher counts. The results of analyzing individual tags also corresponded well with the results of analyzing entire genes.

Fahlgren *et al*. [115] provided another approach for identifying differentially expressed genes from sequencing data sets with replicates by adapting the significance analysis of microarrays (SAM) to sequencing data, a method they call SAM-seq. The differential expression score between the samples incorporates the average abundance across each replicate set for a given sRNA as well as the standard deviation across all samples (from all replicate sets). It also incorporates a small but positive constant to minimize the coefficient of variation for the data set. Therefore, the differential expression score is essentially a *t*-statistic that has been modified to increase inferential power. This approach also uses a *Q*-value to allow for control of the false discovery rate. The power to detect differentially expressed genes (1 - false-negative rate) using this approach increases with the number of replicates as well as with the number of differentially expressed sRNA, but even with five replicates, it still remained in the 75% to 95% range. Conversely, the false discovery rate remained under 5%, even with as few as two replicates.

## Conclusions

The use of next-generation sequencing to analyze small RNA populations is driving a large number of discoveries in many different organisms. The digital nature and the vast sequencing depth afforded by these approaches provide data that is both qualitatively and quantitatively highly informative. The technologies themselves, including read lengths, sequencing depths, cost and methods of library preparation, continue to improve. While standards for these experiments are still lacking, approaches for designing these experiments, preprocessing and normalizing the data and identifying differentially expressed genes continue to develop. To date, most experiments still do not use biological replicates because of cost. The application of the A-C statistic can still allow statistically meaningful conclusions to be drawn from such experiments, but replicates are still ideal. The ability to multiplex samples in single lanes combined with greater sequencing depths will make this financially more feasible, and we expect that in the near future replication will be required as it is for other genomic approaches. While next-generation sequencing is a vast improvement over microarrays for differential gene expression studies, it is not free from bias; the relative levels of different sequences within the same sample do not necessarily represent the biological situation, owing to bias during library preparation. No method is completely free of bias, but it can be reduced by using T4 RNA ligase 2 for adapter ligation, ligation-free library preparation and/or amplification-free sequencing methods. To date, normalization primarily accounts for differences in sequencing depths between libraries, but further experimental study of these biases may enable the biases to be corrected for during normalization. Normalization is still generally done by total linear count scaling, but positive results from RNA-seq and ChIP-seq experiments suggest that quantile-based or nonlinear scaling methods may be more appropriate for sRNA sequencing studies as well because of the abundance of low copy number reads. The issue of multireads complicates all of these analyses. We have attempted to use probability mapping in our studies, but we have found that a single, highly abundant, distinct sequence within a highly conserved region may throw off the apportioning between loci. Probability mapping approaches are also likely affected by sequencing biases, so both issues will need to be accounted for in improved methods.

## Abbreviations

dsRNA: double-stranded RNA; endo-siRNA or esiRNA: endogenous siRNA; exo-siRNA: exogenous siRNA; GMUCT: genome-wide mapping of uncapped transcripts; hc-siRNA: heterochromatic siRNA; LOWESS: locally weighted linear regression; RMA: robust multi-array average; miRNA: microRNA; MPSS: massively parallel signature sequencing; nat-siRNA: natural antisense transcript-derived siRNA; NET-seq: native elongating transcript sequencing; PAP: poly(A) polymerase; PARE: parallel analysis of RNA ends; piRNA: Piwi-interacting RNA; rasiRNA: repeat-associated siRNA; RDR: RNA-dependent RNA polymerase; RNAi: RNA interference; SAM: significance analysis of microarrays; SBL: sequencing by ligation; SBS: sequencing by synthesis; siRNA: small interfering RNA; sRNA: small RNA; ta-siRNA: *trans*-acting siRNA; TMM: trimmed mean of M value; VSN: variance stabilization normalization.

## Competing interests

The authors declare that they have no competing interests.

## Authors' contributions

KPM and MRW prepared this manuscript with input, feedback and advice from BCM. All authors read and approved the final manuscript.
